# The Effect of a Mobile Health Dietary Education Intervention on Ultra-processed Food Consumption in Patients with Type 2 Diabetes: A Randomized Controlled Trial

**DOI:** 10.1016/j.cdnut.2025.107454

**Published:** 2025-04-28

**Authors:** Valeria Cecchini, Linnea Sjöblom, Ylva Trolle Lagerros, Stephanie E Bonn

**Affiliations:** 1 Division of Renal Medicine, Baxter Novum, Department of Clinical Science, Intervention and Technology, Karolinska Institutet, Stockholm, Sweden; 2 Division of Clinical Epidemiology, Department of Medicine, Solna, Karolinska Institutet, Stockholm, Sweden; 3 Department of Medicine, Huddinge, Karolinska Institutet, Stockholm, Sweden

**Keywords:** adults, type 2 diabetes mellitus, dietary patterns, smartphone, processed foods, randomized controlled trial

## Abstract

**Background:**

Globally, there is a significant rise in the consumption of ultra-processed foods (UPFs), which has been associated with negative health outcomes. Mobile health (mHealth) interventions could be used to target dietary intake and reduce UPF consumption.

**Objectives:**

This study aims to evaluate the effect of an mHealth education intervention for healthy eating on UPF consumption in individuals with type 2 diabetes mellitus (T2DM). This study presents the results of an exploratory analysis of data from the Healthy eating using APP technologY (HAPPY) Trial.

**Methods:**

Conducting post-hoc analyses on data from the HAPPY trial, we examined the effect of a 12-week mHealth dietary education intervention. Dietary intake was assessed at baseline and after 3 months using 4-day food records. The NOVA classification was used to quantify UPF consumption. A total of 80 participants (intervention group *n =* 35 and control group *n =* 45) were included in analyses. The intervention effect on UPF consumption was determined by linear regression analysis.

**Results:**

The majority of participants were males (61.3%), the mean age was 63.0 years, and the mean body mass index 29.7 kg/m^2^. In the intervention group, the UPF percentage contribution to the total energy intake was 21.5 interquartile range (IQR): (16.5–31.0) at baseline and 19.7 (IQR: 12.6–30.2) at the 3-month follow-up. In the control group, the UPF percentage contribution was 17.4 (IQR: 13.7–30.5) at baseline and 16.8 (IQR: 9.6–24.9) at the 3-month follow-up. We found no effect of the intervention on UPF consumption.

**Conclusions:**

This exploratory study, based on data from the HAPPY trial, indicates that the mHealth dietary education intervention did not significantly reduce UPF consumption in individuals with T2DM. Interventions specifically targeting UPF intake require further investigation and might provide a promising strategy for addressing this issue in the future.

This trial was registered at clinicaltrials.gov as NCT03784612 (https://clinicaltrials.gov/study/NCT03784612).

## Introduction

Food processing has been an essential part of human history, with methods like drying, freezing, and fermenting being employed since ancient times to preserve food. However, in contemporary society, where food systems have transformed populations’ dietary patterns, convenience, availability, and palatability often dictate consumers’ food choices, resulting in the widespread consumption of ultra-processed foods (UPFs) [1,2]. UPFs are preparations, most often made solely of industrially manufactured ingredients, which are processed and combined using large-scale industrial equipment [3].

The prevalence of UPFs has remarkably reshaped the food landscapes [2]. In Sweden, UPF consumption increased by 142% between 1960 and 2010 [4]. This marked upturn was driven by a higher intake of sugary carbonated drinks and snack foods, including candies and crisps, accompanied by a reduction of unprocessed, minimally processed foods, and processed culinary ingredients [4]. The same trend of increased UPF consumption, alongside the displacement of minimally processed foods and homemade dishes, is mirrored worldwide [3].

The shift in dietary patterns toward a Western diet has significantly influenced the global burden of non-communicable diseases, including cardiovascular disease (CVD), type 2 diabetes mellitus (T2DM), cancer, and obesity [[5], [6], [7], [8]]. Clear causal links between UPFs and these adverse health outcomes are not fully established, but emerging evidence suggests that processing might alter food’s nutritional and chemical properties, potentially affecting health [9]. UPFs often contain additives, contaminants, and compounds like acrylamide and advanced glycation end products, which may increase risk of CVD, T2DM, and obesity [[9], [10], [11], [12]].

Nutritionally, UPFs are energy-dense but lack proteins, dietary fibers, vitamins, minerals, and bioactive compounds, leading to poor dietary quality [13,14]. However, some UPFs, primarily products with specific functional properties, might be fortified [1]. Their palatability can promote overconsumption [15], contributing to overweight and obesity [16,17]. UPFs also tend to have high glycemic loads, which may disrupt hunger and satiety signals, further exacerbating the risk of developing T2DM and obesity [18]. Results from large observational prospective studies suggest a 15% increase in T2DM risk for every 10% rise (by weight) in UPF consumption [19,20]. It is recognized that the majority of new T2DM cases worldwide stem from suboptimal dietary patterns, such as high intake of refined rice, wheat, and sugar-sweetened beverages [21], which may overlap with UPF consumption patterns. Therefore, greater attention should be directed toward understanding and quantifying UPF consumption among individuals at a higher risk of T2DM to aid disease prevention, and among those who have developed T2DM to slow down its progression.

Many interventions have been developed to promote healthy dietary patterns in individuals with T2DM, including the use of mobile health (mHealth) technologies [[22], [23], [24]]. However, these interventions have placed little emphasis on reducing UPF consumption. mHealth solutions offer a promising opportunity for large-scale interventions, given the widespread use of mobile devices [25]. Despite this potential, only a few mHealth interventions have specifically targeted dietary quality [[22], [23], [24]].

This study aimed to assess the effect of a mHealth dietary education intervention, designed to promote healthy dietary patterns, on UPF consumption in individuals with T2DM. Since this is an exploratory analysis of the Healthy eating using APP technologY (HAPPY) trial [26,27], UPF intake was not a prespecified outcome. However, the intervention provided recommendations on dietary factors such as sugar intake, slow and fast carbohydrates, saturated fat, and several other aspects related to UPFs. Thus, it is plausible that an effect on UPF consumption could be observed.

## Methods

### Study design

Data analyzed in the current study stem from the HAPPY trial [26,27]. The latter was conducted as a 2-arm, randomized controlled trial aimed at assessing the impact of a mHealth, application (app)-based intervention, the HAPPY Smartphone App, on dietary intake in individuals with T2DM. Its design, comprehensively described elsewhere [26], is also briefly outlined here.

The HAPPY trial was approved by the Regional Ethical Review Board, Stockholm, Sweden, 2018/652-31; 2018/1094-32; 2018/2393-32; 2020-00591; 2020-07005; 2022-02557-02. The trial was registered on clinicaltrials.gov with the identifier NCT03784612, available at https://clinicaltrials.gov/study/NCT03784612. Participants provided written informed consent before the study’s start.

### Recruitment and randomization

Study participants were recruited from 5 primary healthcare centers in Stockholm, Sweden. Inclusion criteria were diagnosis of T2DM, age of 18 years and older, proficiency in Swedish, access to a smartphone with personal e-identification, and competency in its usage. No specific exclusion criteria were applied. Patients were informed about the study during their routine medical appointment and, those interested in participating, received a phone call from study personnel, who provided further information. Participants were enrolled in the trial between January 2019 and August 2023, when the smartphone app was no longer compatible with the iOS and Android upgrades. Recruitment was also halted during the COVID-19 pandemic in 2020–2021.

Study participants were randomly assigned 1:1 to the intervention group, which utilized the HAPPY Smartphone App alongside standard care, or to the control group, which received only standard care for the first 3 months, with access to the app provided afterward.

### Intervention

The HAPPY Smartphone App was developed to improve dietary intake according to 3 foundational frameworks of behavior change interventions: the health belief model [28], the stages of change model [29], and the social cognitive theory [30]. Behavior change techniques, such as educational information, self-monitoring, goal setting, and feedback, were used to promote consistent dietary modifications.

Healthy dietary patterns were addressed for 12 weeks. The topics covered were: *1*) healthy food patterns, *2*) vegetable intake, *3*) regular eating habits, *4*) sugar intake, *5*) more on vegetable intake, *6*) slow and fast carbohydrates, *7*) whole grains and fibers, *8*) legumes, *9*) saturated fat, *10*) unsaturated fat, *11*) salt intake, and *12*) beverages. Noteworthy in the context of UPFs are the topics about sugar intake, slow and fast carbohydrates, saturated fat, salt intake, and beverages. Each week, the app sent out notifications providing participants with an activity to conduct related to the topic, balanced meal ideas, useful tips, entertaining facts, and a weekly assessment. The app’s features were intended to promote consistent use. However, there was no obligatory commitment, considering that the intervention was self-delivered and self-paced, with a specific emphasis on self-improvement.

In total, the app comprised 136 activities to complete. Adherence to the app’s activity was calculated as the percentage of total completed activities for each participant. Participants who completed ≥80% of the activities were classified as high adherers, whereas those who completed <80% were classified as low adherers.

### Study assessments

Data were collected at baseline (pre-intervention), and at follow-up after 3, 6, and 12 months. The current study used data from the baseline and the 3-month follow-up to assess the intervention’s effect on the consumption of UPFs. Study measurements at baseline and 3 months included a comprehensive web-based questionnaire, which included a 95-item semi-quantitative food frequency questionnaire (FFQ), clinical measurements of height, weight, waist circumference, blood pressure, body composition, hemoglobin A1c (HbA_1c_ which measures the average level of blood sugar over the past 2–3 months), serum lipids, and a 4-day food record, including 3 week days and 1 weekend day [31]. Only participants with food record data at both time points (*n =* 80), i.e., information on UPF intake at both time points, were included in the present analyses.

### Dietary intake and classification of UPF consumption

The HAPPY trial used 2 methods to assess dietary intake: a validated 95-item semi-quantitative FFQ [31,32] and an estimated 4-day food record. In this study, data from the 4-day food records were used to classify participants’ dietary intake and UPF consumption according to the NOVA food classification system [1]. The 4 NOVA groups are unprocessed and minimally processed foods (group 1), processed culinary ingredients (group 2), processed foods (group 3), and UPFs (group 4) [1]. Supplemental Table 1 provides a detailed description of these groups.

Data from the food records were entered into the Dietist Net software package [33], linked to the Swedish Food Agency’s database, by study personnel continuously during data collection. The quality of recordings was checked during entry and, as all records had a daily average between 500 and 3500 kcals, no records were dismissed as implausible. Classification of the 4-day food records according to the NOVA system was conducted under blinded conditions by 1 other researcher. Each dietary record entry was categorized into 1 of the 4 NOVA groups based on its level of industrial processing, applying the guidelines provided by Martinez-Steele et al. [34]. Subsequently, for every participant, the calculation of the total kilocalories (kcal) associated with each NOVA group (absolute intake) was performed to determine the percentage contribution to the total intake (relative intake). To improve the accuracy of estimating participants’ usual intake, the averages of their 4-day food records were calculated, both at baseline and at the 3-month follow-up. Information about energy intake (kcal/day) was sourced from the Dietist Net software package.

To ensure precise classification of food items according to the NOVA classification, information about food ingredients is important. To address this, we examined the ingredient lists of commonly consumed composite dishes in Sweden when participants had not recorded the brand of the packaged food they ate. When brand information was recorded, the food was searched for on Swedish supermarket websites, and its ingredient list was assessed.

To minimize classification uncertainty and potential misclassification, we reclassified all uncertain food items to create both a lower-bound and an upper-bound classification scenarios, as recommended by Martinez-Steele et al. [34]. In the most conservative approach, uncertain items requiring additional information like brand name, or further specifications to confidently assign them to a NOVA group, were classified into the lower degree of processing, e.g., processed foods. Conversely, in the least conservative approach, these items were classified into the higher degree of processing, e.g., UPFs.

### Power calculations

The HAPPY trial aimed to recruit 200 participants, to observe a clinically significant change of 4 mmol/mol in HbA_1c_ and to provide 80% power at a 5% significance level, accompanied by a 20% dropout rate.

### Statistical analyses

Descriptive statistics including mean and SD for continuous variables and *n* and percentages for categorical variables were used to present the baseline characteristics of the study population. Within-group changes in consumption of NOVA food groups between baseline and the 3-month follow-up were assessed using the Wilcoxon Signed-Rank Test, due to the non-normality of the data. To determine the intervention’s effect, differences in absolute energy intake from UPFs from baseline to the 3-month follow-up, between the intervention and control group, were analyzed using linear regression models adjusted for baseline values [35]. Sensitivity analyses were performed comparing the most conservative classification approach to the least conservative one. Participants were categorized into high and low-adherence groups based on their engagement with the app, allowing for an analysis of changes in UPF consumption among those participants who received the intervention. However, as the majority of the participants in the intervention group were high adherers, stratified analyses could not be performed. All analyses were performed using STATA version 17.0 (Stata Corporation) with a significance level of 0.05.

## Results

**FIGURE 1:**
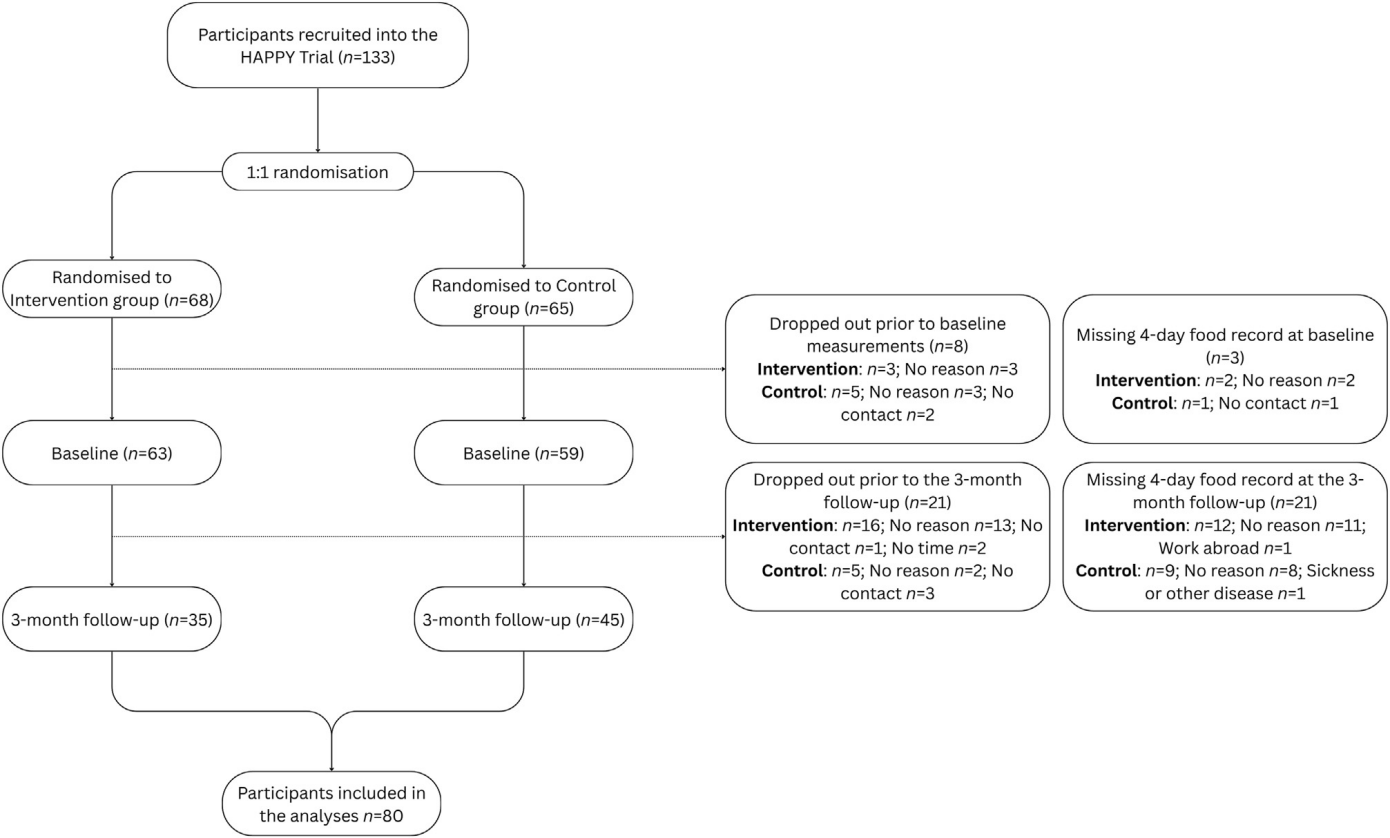
Flow diagram of participants included in the HAPPY trial and in the analyses of ultra-processed food consumption in this study. HAPPY, Healthy eating using APP technologY.

Figure 1 outlines participants included in the trial and in the analyses of UPF consumption in this study. In total, 133 males and females were recruited. Due to a high dropout rate and missing data from food records, we included 80 participants who completed the 4-day food records at baseline and at the 3-month follow-up. The baseline characteristics of these participants are outlined in Table 1. The majority (61.3%) were males, with a mean age of 63.0 years. Most of the participants, 73.8%, used oral medication for T2DM, such as metformin, and 65% used blood pressure medication. There were no statistically significant differences in baseline characteristics between participants with complete data from baseline and the 3-month follow-up (*n =* 80) compared with those with missing data at any of the 2 time points (*n =* 53) (*P* > 0.05).

**TABLE 1:** Baseline characteristics of the study population (*n =* 80), divided by intervention (*n =* 35), and control group (*n =* 45).

Characteristics	Total (*n =* 80)		Intervention (*n =* 35)		Control (*n =* 45)	
	Mean	(SD)	Mean	(SD)	Mean	(SD)
Age (y)	63.0	(10.5)	63.5	(9.2)	62.4	(11.4)
BMI (kg/m^2^)	29.7	(4.9)	29.5	(5.6)	29.8	(4.3)
HbA_1c_ (mmol/mol)	49.8	(9.8)	49.9	(10.4)	49.7	(9.4)
Total energy intake (kcal/d)	1927	(471)	1922	(496)	1930	(456)

	*n*	(%)	*n*	(%)	*n*	(%)

Sex						
Female	31	(38.8)	13	(37.1)	18	(40.0)
Male	49	(61.3)	22	(62.9)	27	(60.0)
Prior medication use (yes;%)						
Blood pressure medication	52	(65.0)	21	(60.0)	31	(69.0)
Insulin	15	(18.8)	6	(17.1)	9	(20.0)
Oral medication for T2DM	59	(73.8)	24	(68.6)	35	(77.8)
Medication for blood lipids	45	(56.3)	18	(51.4)	27	(60.0)

Abbreviations: HbA_1c_, hemoglobin A1c; T2DM, type 2 diabetes mellitus.

Table 2 displays the changes in consumption of NOVA groups within the intervention and control groups from baseline to the 3-month follow-up, by applying both classification methods. No statistically significant differences in the percentage of energy from UPFs (relative intake) were observed between baseline (median 21.5; IQR: 16.5–31.0) and 3 months [19.7 (12.6–30.2)] in the intervention group (*P =* 0.39). There was a rise in the percentage contribution of group 1 foods to the total energy intake (TEI), although this was not statistically significant [baseline: 33.0 (24.8–39.2); 3 months: 35.1 (25.2–40.1)]; *P =* 0.27. The control group exhibited similar results, showing a non-statistically significant increase in consumption of group 1 foods [baseline: 32.5 (27.0–39.9); 3 months: 39.4 (29.0–45.7)]; *P =* 0.08, accompanied by a statistically significant reduction in the consumption of UPFs from baseline [17.4 (13.7–30.5)] to the 3-month follow-up [16.8 (9.6–24.9)]; *P =* 0.02.

**TABLE 2:** Energy intake (kcal) of each NOVA group for the intervention (*n =* 35) and control group (*n =* 45) from baseline to the 3-month follow-up, by applying both classification methods.

NOVA	Most conservative classification				*P*^1^
	Intervention (*n =* 35)				
	Baseline		3 month		
	Absolute (kcal)	Relative (%)	Absolute (kcal)	Relative (%)	
	Median (IQR)	Median (IQR)	Median (IQR)	Median (IQR)	
Group 1	647.9 (508.0–731.8)	33.0 (24.8–39.2)	586.1 (422.9–688.7)	35.1 (25.2–40.1)	0.27
Group 2	111.1 (48.4–146.9)	5.9 (2.4–8.2)	75.0 (40.1–173.4)	6.2 (2.1–9.2)	0.87
Group 3	679.4 (507.0–851.1)	36.9 (27.4–44.8)	666.5 (476.6–780.7)	35.6 (29.7–41.7)	0.77
Group 4	369.9 (250.5–631.2)	21.5 (16.5–31.0)	367.6 (176.8–544.8)	19.7 (12.6–30.2)	0.39

NOVA	Control (*n =* 45)				
	Baseline		3 month		
	Absolute (kcal)	Relative (%)	Absolute (kcal)	Relative (%)	
	Median (IQR)	Median (IQR)	Median (IQR)	Median (IQR)	
Group 1	656.8 (540.5–795.4)	32.5 (27.0–39.9)	658.4 (545.2–815.0)	39.4 (29.0–45.7)	0.08
Group 2	108.4 (55.4–187.0)	5.2 (3.0–9.1)	105.2 (64.4–143.7)	6.0 (3.6–8.0)	0.84
Group 3	645.1 (510.6–848.9)	33.9 (29.3–44.2)	603.4 (471.8–920.5)	36.1 (26.9–47.7)	0.93
Group 4	357.6 (217.3–620.5)	17.4 (13.7–30.5)	300.4 (193.1–436.6)	16.8 (9.6–24.9)	0.02

NOVA	Least conservative classification				*P*^1^
	Intervention (*n =* 35)				
	Baseline		3 month		
	Absolute (kcal)	Relative (%)	Absolute (kcal)	Relative (%)	

Median (IQR)	Median (IQR)	Median (IQR)	Median (IQR)
Group 1	647.9 (508.0–731.8)	33.0 (24.8–39.2)	586.1 (422.9–688.7)	35.1 (25.2–40.1)	0.27
Group 2	111.1 (48.4–146.9)	5.9 (2.4–8.2)	75.0 (40.1–173.4)	6.2 (2.1–9.2)	0.87
Group 3	507.0 (354.8–641.9)	25.9 (19.3–37.6)	412.6 (275.1–577.3)	23.8 (18.1–30.5)	0.16
Group 4	606.3 (296.4–843.7)	34.2 (20.8–40.4)	598.8 (375.2–781.9)	33.1 (24.0–45.3)	0.61

NOVA	Control (*n =* 45)				
	Baseline		3 month		

	Absolute (kcal)	Relative (%)	Absolute (kcal)	Relative (%)	

Median (IQR)	Median (IQR)	Median (IQR)	Median (IQR)
Group 1	656.8 (540.5–795.4)	32.5 (27.0–39.9)	658.4 (545.2–815.0)	39.4 (29.0–45.7)	0.08
Group 2	108.4 (55.4–187.0)	5.2 (3.0–9.1)	105.2 (64.4–143.7)	6.0 (3.6–8.0)	0.84
Group 3	545.3 (335.3–667.8)	27.8 (21.1–34.4)	458.5 (392.8–582.4)	25.5 (21.9–32.7)	0.49
Group 4	542.0 (342.6–792.4)	30.4 (19.1–40.4)	507.8 (314.8–649.9)	29.2 (18.5–35.4)	0.34

Abbreviations: Group 1, unprocessed and minimally processed foods; group 2, processed culinary ingredients; group 3, processed foods; group 4, NOVA; UPFs; UPF, ultra-processed food.

1 *P* values obtained by Wilcoxon Signed-Rank Test comparing relative intake at baseline and 3 months.

By applying the least conservative classification method, the sensitivity analyses showed that in the intervention group, UPF energy contribution was 34.2% (20.8–40.4) of the TEI at baseline, decreasing to 33.1% (24.0–45.3) at 3 months (*P =* 0.61). In the control group, UPF consumption decreased from 30.4% (19.1–40.4) at baseline to 29.2% (18.5–35.4) at 3 months (*P =* 0.34).

In the intervention group, the share of energy from UPFs increased from 21.5% (16.5–31.0) to 34.2% (20.8–40.4) at baseline, and from 19.7% (12.6–30.2) to 33.1% (24.0–45.3) at 3 months, when comparing the most conservative to the least conservative classification methods, respectively. In the control group, the UPF energy share rose from 17.4% (13.7–30.5) to 30.4% (19.1–40.4) at baseline, and from 16.8% (9.6–24.9) to 29.2% (18.5–35.4) at 3 months, under the same classification criteria.

Table 3 shows the results of the linear regression models addressing the effect of the intervention. No statistically significant differences were observed in the effect of the intervention on the consumption of NOVA food groups.

**TABLE 3:** Mean values and SD of the differences between baseline and the 3-month follow-up of the absolute intakes (kcal) for the study groups, by applying both classification methods.

Most conservative classification						
	Intervention (*n =* 35)		Control (*n =* 45)			
	Difference		Difference		Model estimates	
	Mean	(SD)	Mean	(SD)	*β*^1^	(95% CI)
Group 1	45.7	(176.7)	−17.2	(207.0)	74.2	(−1.6, 149.9)
Group 2	16.6	(115.9)	13.8	(104.9)	4.0	(−34.5, 42.5)
Group 3	30.3	(280.5)	−4.1	(319.3)	38.0	(−89.8, 165.8)
Group 4	84.2	(292.2)	108.9	(269.7)	−42.5	(−135.4, 50.4)

Least conservative classification						

	Mean	(SD)	Mean	(SD)	*β*	(95% CI)

Group 1	45.7	(176.7)	−17.2	(207.0)	74.2	(−1.6, 149.9)
Group 2	16.6	(115.9)	13.8	(104.9)	4.0	(−34.5, 42.5)
Group 3	76.4	(235.4)	34.5	(233.4)	48.3	(−42.6, 139.2)
Group 4	38.2	(368.8)	70.3	(319.8)	−51.6	(−174.3, 71.1)

Intervention’s effect is shown as difference in changes (*β* and 95% CI) between the study groups.

Abbreviations: CI, confidence interval; group 1, unprocessed and minimally processed foods; group 2, processed culinary ingredients; group 3, processed foods; group 4, UPF; UPF: ultra-processed food.

1 Linear regression for continuous outcomes adjusted for baseline values.

Adherence analyses showed that high adherers were *n =* 33 (94.3%) and low adherers were *n =* 2 (5.7%), thus preventing the possibility of conducting stratified analyses.

## Discussion

We found no statistically significant effect of a 12-week mHealth dietary education intervention, designed to promote healthy dietary patterns, on absolute energy intake from UPFs in individuals with T2DM. To our current knowledge, this is the first study to explore the effects of a mHealth intervention on UPF consumption.

The lack of an observed effect on UPF consumption in the HAPPY trial could be explained by the study being underpowered to detect changes in UPF intake. It is a limitation that power was not calculated for detecting a change in dietary intake and UPF consumption. Another explanation for the lack of an effect is the absence of a specific focus on UPFs within the intervention. However, several topics covered in dietary education were closely related to UPFs, such as sugar intake, slow and fast carbohydrates, and saturated fat. Current results indicate an increase in unprocessed and minimally processed foods in the intervention group, aligning with the specific topics covered in the app, such as vegetable intake (weeks 2 and 5), whole grains and fibers (week 7), and legumes (week 8). This suggests a need for a more tailored approach within the app content to explicitly address UPF consumption if the aim is to decrease it. Altering behaviors is difficult and interventions are more likely to yield noticeable results when they are tailored to the specific behavior [36]. Since the introduction of the final version of the NOVA food classification system in 2019, a few different apps targeting UPF consumption have been developed. Some of these apps have aimed at assisting individuals in identifying UPFs while shopping, utilizing features like barcode scanning, e.g., Open Food Facts [37] and Processed [38].

Generally, another possible explanation for a lack of an intervention effect is low adherence to an intervention. Low user engagement and adherence have been a limitation in previous mHealth interventions and may lead to null effects [39]. However, adherence to the HAPPY App in our study was high and nearly all participants in the intervention group (94.3%) were classified as high adherers, meaning that they had completed ≥80% of all activities available in the app. Therefore, low adherence is likely not a limiting factor to the effectiveness of our intervention. However, it is important to note that although adherence was high, the dropout rate was also high.

Of particular interest in the current study are the findings regarding the contribution of UPFs to the TEI. A recent study indicated that > 41% of the TEI for adult males and 44% for adult females in Sweden were derived from UPFs [40]. This is a remarkably higher proportion compared with the one observed in this study. We found UPF dietary shares of 21.5% for the intervention and 17.4% for the control group. After reclassifying uncertain food items, the UPF energy share was 34.2% for the intervention group and 30.4% for the control group. Both figures remain below the levels reported in the general Swedish population [40]. This might be because individuals with T2DM usually receive comprehensive dietary education post-diagnosis, which contributes to higher health literacy [41]. Furthermore, higher health literacy has been associated with lower consumption of UPFs, as individuals with greater nutrition knowledge and food literacy tend to make healthier food choices [42].

Unexpectedly, our findings showed a statistically significant, yet small, decrease in UPF consumption within the control group from baseline to the 3-month follow-up. Several factors may account for this. Participants’ heightened awareness of their behaviors, simply by enrolling in a diet study, may have led them to make conscious efforts toward dietary improvement, even without the app-based intervention. External factors not accounted for in the analyses, such as access to health-related information or resources facilitating dietary changes, could have further influenced their choices and behaviors. Furthermore, it is possible that this decrease reflects changes in self-reporting behavior than actual intake, as dietary self-reports in intervention studies are susceptible to measurement error and biases, including social desirability and increased self-monitoring [43].

### Strengths and limitations

The present study exhibits several strengths and acknowledges certain limitations that warrant consideration. The randomized study design is a considerable strength, enhancing the robustness of the findings. The study’s inclusion of a representative sample of individuals with T2DM from the Swedish population, encompassing both sexes, is an additional significant strength. The BMI and HbA_1c_ distribution within the study population closely resembled the average seen in Swedish primary care [44]. Recruitment from primary care centers can further increase the generalizability of the findings. However, it is worth noting that the 5 primary care centers chosen for recruitment were all located in relatively higher-income areas of Stockholm, Sweden, potentially introducing some bias in participant selection. Moreover, the inclusion criteria regarding fluency in Swedish might have further excluded recent immigrants from participating, thereby reducing the generalizability of the study’s results. Notably, males constituted 61.3% of the total sample, which aligns with the sex distribution observed in the Swedish primary care settings [44]. The latter differs from the average trend observed in mHealth interventions, which often involves predominantly females, typically with higher educational backgrounds [45].

Although dietary records are often considered one of the more accurate self-report methods for assessing food intake [46] they are not without limitations. As subjective methods, they are influenced by individuals’ accuracy in reporting meals. Biomarker-based validation studies suggest that dietary records tend to provide more precise short-term estimates compared with FFQs, which assess intake over a longer period of time and may be more prone to recall bias [46,47]. However, like all self-reported dietary assessment tools, dietary records remain susceptible to underreporting and measurement errors [48]. Another limitation of dietary records concerns infrequently consumed foods, including episodically consumed foods, that may go undocumented [48].

The current study faces limitations, including limited statistical power, resulting from a small sample size and missing dietary record data. Furthermore, a higher number of participants discontinued their involvement in the intervention group, compared with the control group, resulting in missing data at the 3-month follow-up. If those who discontinue participation in a trial differ in characteristics and experiences compared with those who remain in the intervention, this may bias the results. However, we found no statistically significant differences in characteristics between participants with complete data and those without.

Given the nature of the intervention, blinding was not feasible, which may partly explain the observed change in UPF consumption between baseline and 3 months seen in the control group. Since participants were aware that they were part of a dietary study, this could have led to changes in their dietary behavior, such as increased attention to their food choices, influencing UPF consumption.

The decision to utilize the NOVA food classification system stemmed from its recognized specificity, consistency, clarity, comprehensiveness, and practicality in identifying the level of food processing [49,50]. Nevertheless, there are known limitations to the NOVA system. Some of these highlight that the level of food processing does not necessarily indicate the food’s nutritional value. Although many foods classified as UPFs are widely recognized as such, it is worth acknowledging that several food items typically regarded as healthy, such as packaged whole grain bread, fruit yogurts, and plant-based analogs, also fall under the UPF category [51]. Further research is required to determine whether the processing techniques used or the ingredients and the presence of food additives, make these products as harmful as other UPFs [52]. To ensure precise classification of data in our study, a thorough analysis of ingredient formulations for commonly consumed foods in Sweden was performed during food classification. This process aimed to address any potential limitations arising from missing brand information in the 4-day food records. Although the lack of brand details may have impacted the reliability of the findings, the current results are strengthened by close adherence to the guidelines for applying the classification [34]. Recent evidence from metabolomics studies provides valuable insight into objective methods to identify UPF consumption, considering that there is significant variability in dietary patterns that include these foods [53]. Thus, metabolomic profiling might serve as an objective complement to the NOVA classification in future studies.

Designing mHealth interventions targeting UPF intake requires a thorough understanding of how people consume UPFs. Thus, future studies are needed to explore patterns of UPF consumption among the general population and in individuals with diseases, such as T2DM, where diet is of particular importance for preventing complications.

In conclusion, we found no effect of a 3-month mHealth dietary education intervention, designed to promote healthy dietary patterns, on UPF consumption in individuals with T2DM. Nevertheless, tailored mHealth interventions aiming to reduce UPF consumption might be a promising strategy to address this issue in the future.

## Author contributions

The authors’ responsibilities were as follows – SEB: principal investigator of the HAPPY trial; SEB, YTL: responsible for the resources and funding acquisition; LS: data collection; VC, SEB: conceptualization of the present study and wrote the first draft of the manuscript; VC: performed classification of the 4-day food records according to the NOVA food classification system and the analyses included in this study and wrote the first draft of the manuscript; and all authors: read, provided critical feedback on, and approved the final manuscript.

## Data availability

Data described in the manuscript, code book, and analytic code will be made available on request pending application and approval from the principal investigator (SEB).

## Funding

This research was supported by the Strategic Research Area Health Care Sciences (SFO-V) at Karolinska Institutet (SEB), Karolinska Institutet Doctorial funding (SEB), grants provided by Region Stockholm (NSV project) (YTL), and funding from the regional agreement between Stockholm County Council and Karolinska Institutet, clinical research appointment (YTL). The funders had no role in the study design, data collection, analysis, interpretation, manuscript preparation, or in the decision to publish the findings.

## Conflict of interest

The authors report no conflicts of interest.
