# Supplementary material for: The Effect of a Mobile Health Dietary Education Intervention on Ultra-processed Food Consumption in Patients with Type 2 Diabetes: A Randomized Controlled Trial
Source: Curr Dev Nutr. 2025 Apr 28;9(6):107454. doi: 10.1016/j.cdnut.2025.107454 (PMC12162007; doi:10.1016/j.cdnut.2025.107454)
Supplement: Multimedia component 1 [file mmc1.docx]

**Supplementary Table 1**. The NOVA classification based on the nature, degree, and purpose of industrial processing (1,53).

| **Nova groups** | **Characteristics** | **Examples** |
| --- | --- | --- |
| Unprocessed and minimally processed foods | Unprocessed foods are consumable elements of plants, animals, fungi, etc. post-extraction from nature, whereas minimally processed foods are unprocessed foods that sustained limited industrial processing, e.g. powdering, pasteurisation. These processes primarily serve to prolong the microbiological profile of unprocessed foods, allowing for extended storage. | Fresh, squeezed, dried fruits, vegetables, whole grains and rice, beans, mushrooms, and nuts. Meat and poultry, eggs, fish. Pasta, couscous, and polenta from flours. Herbs, teas, coffee, and water. |
| Processed culinary ingredients | Processed culinary ingredients are derived from Group 1 foods or extracted from nature through techniques such as dewatering and grinding. These techniques are used to produce the seasonings for Group 1 foods. Additives are infrequently found in this group. | Vegetable oils from seeds, nuts and fruits, animal fats, sugar, honey, and salt. |
| Processed foods | Food items created by combining one or more Group 2 items to Group 1 foods through techniques such as non-alcoholic fermentation and bottling. Processing aims at improving the longevity and sensory attributes of the foods. This group excludes any additives with cosmetic functions. | Bottled vegetables and legumes. Curated meat and fish. Freshly made breads and cheeses, featuring only Group 1 and Group 2 foods. |
| Ultra-processed food | Industrial formulations of ingredients with limited or no culinary purpose. Group 1 foods are present in minimal to negligible quantities. Industrial techniques utilised in the production of UPFs include moulding, extrusion, and pre-frying. Furthermore, this group contains additives serving cosmetic purposes, such as sweeteners, thickeners, carbonating, foaming, bulking, and gelling agents. Processing aims at creating hyper-palatable, profitable, convenient products, liable to displace all other NOVA food groups. | Carbonated sugary beverages, packaged sweet and salty snacks, chocolates, confectionery products, margarines, and sauces. Ready-to-eat/heat foods, plant-based alternatives, and meal replacement shakes. |
